# Cryoballoon Ablation for Persistent Atrial Fibrillation in a Patient with a Left Pneumonectomy

**DOI:** 10.19102/icrm.2021.121201

**Published:** 2021-12-15

**Authors:** Vijaywant Brar, Huzaifa Ahmad, Manavotam Singh, Susan O’Donoghue, Seth J. Worley

**Affiliations:** ^1^Division of Cardiac Electrophysiology, MedStar Heart and Vascular Institute, MedStar Washington Hospital Center, Washington, DC, USA

**Keywords:** Atrial fibrillation, cryoballoon ablation, pneumonectomy, pulmonary vein isolation

## Abstract

Pulmonary vein (PV) isolation (PVI) is the most important component of catheter ablation of atrial fibrillation (AF) and can be achieved by radiofrequency or cryoballoon ablation (CBA). The CBA system has shown excellent efficacy and safety in a number of clinical trials and is independent of the PV anatomy. However, pneumonectomy can significantly alter the anatomy posing a challenge to CBA. Few cases of PVI accomplished by CBA have been described in patients with lobectomy, but none in the pneumonectomy population. We describe a case of successful CBA for paroxysmal AF in a patient with a left total pneumonectomy.

## Background

Radiofrequency (RF) and cryoballoon ablation (CBA) have become standard treatment options for atrial fibrillation (AF). Both the 2012 American College of Cardiology/American Heart Association/Heart Rhythm Society and the 2020 European Society of Cardiology guidelines for the diagnosis and management of AF recommend catheter ablation (class I level of evidence A) for patients with symptomatic paroxysmal AF refractory or intolerant to anti-arrhythmic drugs.^1^ Pulmonary vein (PV) isolation (PVI) is the cornerstone of catheter ablation of AF, and CBA has become an increasingly utilized strategy for achieving PVI in patients with AF.^2^ Previous studies have shown that the efficacy of CBA is independent of the PV anatomy.^3^ Few cases of successful CBA have been reported in patients with a history of pulmonary lobectomies, but none exist in those with a history of total pneumonectomy.^4^ A pneumonectomy can result in significant anatomical alterations, including changing the position of the heart and large vessels, posing a challenge to PVI.^5^ We describe a unique case of successful CBA in a patient with persistent AF and a history of left pneumonectomy.

## Case presentation

A 76-year-old man with a past medical history of hypertension, coronary artery disease (focal stenosis of the first obtuse marginal branch, which was medically treated), and non-tuberculous mycobacterial left lung infection complicated by chronic severe left bronchiectasis necessitating a left pneumonectomy was referred to our institution for the management of symptomatic persistent AF. He was first diagnosed with persistent AF about a year prior and required cardioversion to normal sinus rhythm. He remained in normal sinus rhythm for about six months, followed by recurrence of AF. At that time, he was started on sotalol therapy, which was discontinued due to excessive fatigue. He was not a candidate for amiodarone or other anti-arrhythmic drugs due to his chronic non-tubercular mycobacterial lung infection and potential drug interactions, notably QT prolongation, with his chronic antibiotic therapy. Therefore, the decision was made to proceed with AF ablation.

**Figure 1:**
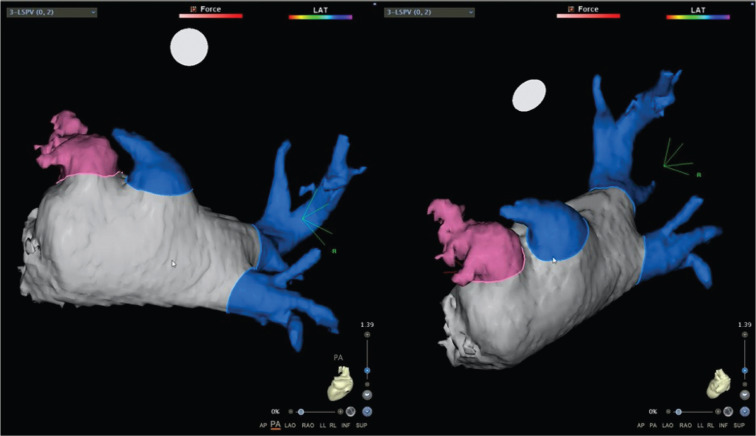
Cardiac CT in the posteroanterior and left lateral projections. The left atrial appendage is colored pink, and the veins are colored blue.

**Figure 2:**
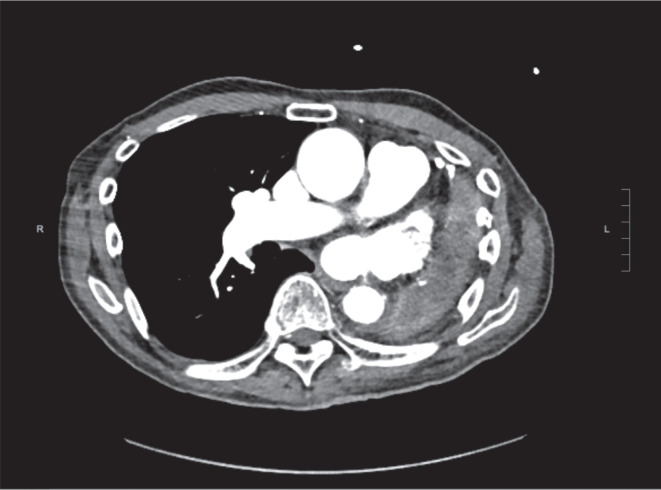
Cardiac CT scan demonstrating a left mediastinal shift in the patient.

As part of routine pre-procedural planning, the patient underwent contrast computed tomography (CT) imaging of the heart, which excluded an intracardiac thrombus **(Figure 1)** and demonstrated a left mediastinal shift due to his pneumonectomy **(Figure 2)**. Intracardiac echocardiography (ICE) was also performed to help define the anatomy of the interatrial septum and guide transseptal puncture. Transseptal puncture was more challenging in this patient due to the rotation of the heart, but imaging helped to overcome this challenge. Pre-interventional imaging also helped define the PV anatomy. The patient had a remnant left common PV stump as a result of his history of left pneumonectomy and two widely patent right-sided PVs. The length of the left common PV stump was noted to be around 30 mm. The size of the veins is given in **Table 1**. Anchoring the CBA catheter in the PV stump can be difficult if the length of the pulmonary stump is short. After carefully reviewing the anatomy, it was concluded that the length of the left common PV remnant was sufficient to attempt CBA.

**Figure 3:**
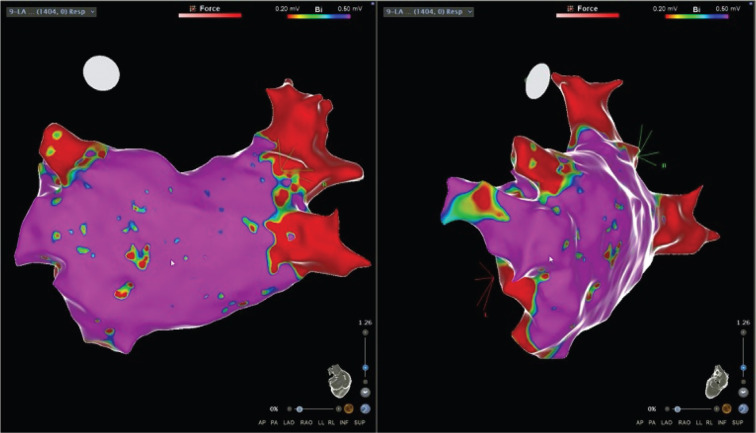
Pre-ablation voltage map using the CARTO^®^ electroanatomic mapping system in the posteroanterior and left lateral projections. The left common vein stump and the two right-sided veins all appear to be electrically active.

**Figure 4:**
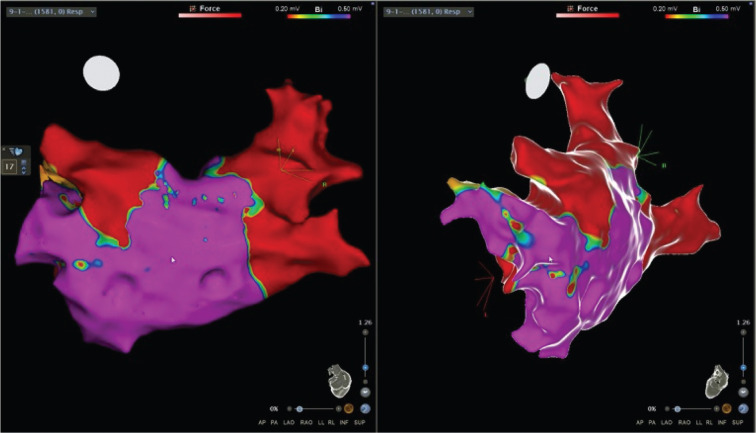
Postablation voltage map using the CARTO^®^ electroanatomic mapping system in the posteroanterior and left lateral projections. All the veins appear to be electrically isolated.

For the CBA, the patient was placed under general anesthesia and an esophageal temperature probe was inserted for close monitoring. We first performed voltage mapping of the left atrium using the PentaRay^®^ mapping catheter (Biosense Webster, Diamond Bar, CA, USA) and the CARTO^®^ electroanatomic mapping system (Boston Scientific, Natick, MA, USA). The latter demonstrated electrical activity in the left common PV stump and the two right-sided PVs **(Figure 3)**. We then advanced the Achieve catheter (Medtronic, Minneapolis, MN, USA) to the left common PV stump. Thereafter, we were able to successfully occlude the left common PV stump using a 28-mm Arctic Front™/Achieve cryoballoon catheter system (Medtronic) and achieve electrical isolation. Then, we successfully performed CBA of the right-sided veins while monitoring the phrenic nerve function. Each of the PVs was frozen once if isolation was seen by 30 seconds, and, if not seen, the veins were frozen twice. Local temperature, esophageal temperature, time to −30°, and thawing were monitored during each isolation procedure. Post-CBA, voltage mapping of the left atrium demonstrated isolation of all three PVs **(Figure 4)**. He was in normal sinus rhythm after the procedure and was discharged home. Unfortunately, at the one-month follow-up visit, he was found to have recurrence of AF. The patient was unable to tolerate atrioventricular (AV) nodal blocking agents due to hypotension, and anti-arrhythmic drugs were contraindicated due to harmful interactions with chronic antibiotic therapy for non-tubercular mycobacterial infection. After patient-centered discussion, he was treated with an AV node ablation and permanent pacemaker implantation.

## Discussion

PVI is the mainstay of treatment in the catheter ablation of AF. PVI eliminates the electrophysiological triggering activity of muscular sleeves at the conjunctional points between the left atrium and the PVs.^6^ The landmark FIRE AND ICE trial showed that AF ablation using cryoballoon is as effective as using the contact force-guided RF energy.^7^ These modalities primarily differ in their side-effect profile. RF ablation is associated with a higher incidence of atrioesophageal fistula, whereas cryoablation is associated with a greater risk of phrenic nerve injury.^7^

Pulmonary venous anatomy is more variable than the anatomy of the pulmonary arterial system. Furthermore, developmental anomalies such as a single common left PV are commonly encountered in clinical practice.^8^ Radiological studies assessing the prevalence and characterization of PV variants in patients with AF revealed anatomical variants in over 24% of the cases, with a high prevalence of left common trunks and supernumerary right PVs.^9^ Current ablation approaches mainly target the antral region of PVs, but the use of a newer and larger 28-mm second-generation balloon in CBA has reduced constraints from anatomical variations, including larger PVs, in patients with AF.^10,11^ Khoueiry et al. demonstrated that, regardless of the PV anatomy, both RF and CBA had similar efficacy and safety profiles at mid-term follow-up of AF ablation.^3^ Furthermore, in the FIRE AND ICE trial, cryoablation was comparable to RF in terms of efficacy in patients not selected on the basis of their anatomy.^6^

There is limited literature on the impact of pneumonectomy on outcomes with CBA. The previous studies did not include patients with a history of pneumonectomy, which can significantly alter the PV anatomy. Kanmanthareddy et al. demonstrated that PVI of PV stumps in post-lobectomy patients with AF was possible using RF ablation, but the study did not include CBA.^12^ Although case reports of patients with pulmonary lobectomies undergoing successful CBA for paroxysmal AF have been documented, lobectomies usually do not lead to significant anatomical changes in cardiac and other thoracic structures.^4,13^ In contrast, a left pneumonectomy can result in cardiac rotation due to mediastinal shifting **(Figure 4)**, and a right pneumonectomy can result in a lateral shift of the heart.^5^ Cardiac rotation causes difficulties in angiographic visualization of PVs, which is an important step in achieving effective PVI.^4^ After a pneumonectomy, remnant PV stumps typically have lengths of more than 20 mm, whereas muscular sleeves typically have lengths below 10 mm but can reach up to 25 mm inside the PVs.^14^ Thus, remnant PV stumps are electrically active and frequently remain the sites of active firing, underlining the importance of PVI of the stumps.^15^ In a study investigating the challenges of PVI in 19 patients with a prior pulmonary lobectomy or pneumonectomy, Fink et al. achieved complete PV visualization in only three (37.5%) patients with a prior pneumonectomy, resulting in incomplete PVI and consequently high rates of recurrent arrhythmias. Of the four patients in the study who underwent a CBA, only one had a history of a total pneumonectomy. In this patient, the left PV stumps could not be visualized angiographically during the index procedure, hence only the right PVs were isolated. The authors did not employ preoperative imaging due to institutional limitations and concluded with a strong recommendation for pre-interventional imaging techniques such as magnetic resonance imaging in patients with a previous pneumonectomy to achieve complete PVI.^4^

The modality of choice for patients with a previous pneumonectomy remains unclear and is often institution-dependent. We are a high-volume center that uses CBA as the modality of choice for patients with AF. Our practice is supported by a recent study by Mörtsell et al., who studied the second-generation cryoballoon and the irrigated RF energy with regard to outcomes and safety. The study demonstrated lower re-ablation rates and shorter procedure times with CBA as compared to RF ablation.^16^ Our experience with RF showed higher rates of post-ablation atypical flutter compared to CBA. The difference in rates of atypical flutter was not investigated in the FIRE AND ICE trial, and further studies are needed to explore this outcome. We routinely utilize preoperative imaging, including cardiac CT angiography and ICE, to delineate the variable relationship between the left atrium, PVs, and surrounding structures and to exclude a left atrial appendage thrombus. We also routinely use the pre-ablation voltage map prior to AF ablation using cryoballoon or RF in all patients in our institution. In this patient who had previously undergone a left pneumonectomy, the left common PV was clipped and ligated with a residual stump. After reviewing the anatomy of the left common PV remnant based on cardiac CT, ICE, and the fast anatomical map, we determined that the length of the PV stump was sufficient to allow effective anchoring of the cryoballoon using the Achieve catheter, and so a decision was made to proceed with CBA. Hence, we were able to successfully achieve complete PVI in all three PVs using the CBA catheter system **(Figures 1, 3, and 4)**.

This case highlights the importance of careful selection of patients for CBA, based upon meticulous review of their pre-interventional cardiac CT and intraprocedural imaging. Preprocedural imaging in such patients is helpful for delineating the PV anatomy; identifying potential technical difficulties; and, most importantly, selection of therapy, ie, RF ablation versus CBA.^17^ To the best of our knowledge, our case is the first to demonstrate that AF CBA can be successfully performed in patients with prior total pneumonectomy.

## Conclusion

This report illustrates the successful CBA of persistent AF in a patient with a prior left total pneumonectomy. The choice of modality for AF ablation in patients with a pneumonectomy should be individualized based on their preoperative and intraoperative cardiac imaging. Larger studies are needed to determine the differences in outcomes using RF ablation or CBA in this population.

## Figures and Tables

**Table 1:** Size of the PVs

	Diameter (mm)	Length (mm)
Left common vein stump	23.6	29.5
RSPV	20.1	–
RIPV	20.8	–

CT: computed tomography; RIPV: right inferior pulmonary vein; RSPV: right superior pulmonary vein.

Measurements were taken from cardiac CT images.
